# Impact of COVID-19 on neglected tropical diseases in Zambia: a qualitative study of stakeholder perspectives

**DOI:** 10.1186/s40249-026-01491-0

**Published:** 2026-08-25

**Authors:** Caitlin Brigid Butala, Jenna Fyfe, Noreen Machila, Susan Christina Welburn

**Affiliations:** 1 Zhejiang University-University of Edinburgh Joint Institute, Zhejiang University, International Campus, 718 East Haizhou Road, Haining, 314400 China; 2 Disease Control Department, School of Veterinary Medicine, University of Zambia, Po box 32379, Lusaka, 10101 Zambia; 3 IRR Edinburgh Medical School, College of Medicine and Veterinary Medicine, University of Edinburgh, 1 George Square, EH8 9JZ Edinburgh, United Kingdom

**Keywords:** Neglected tropical diseases, COVID‑19, Qualitative study, Zambia, Health system, One Health, Funding, Mass drug administration

## Abstract

**Background:**

Neglected tropical diseases (NTDs) remain a major public health challenge in Zambia and other low‑ and middle‑income countries, and there is concern that the COVID‑19 pandemic has further disrupted progress towards control and elimination targets. This study explored how COVID‑19 affected NTD programmes, funding, and partnerships in Zambia from the perspective of national‑level stakeholders.

**Methods:**

We conducted a qualitative case study using semi‑structured interviews with key informants involved in NTD policy, programming, or surveillance in Zambia. Fourteen participants were purposively sampled from government ministries, the Zambia National Public Health Institute, international organisations, and academia. Interviews were conducted in person, audio‑recorded, transcribed verbatim, and anonymised. Data were analysed using inductive thematic analysis, with codes developed from the transcripts and iteratively grouped into themes through constant comparison.

**Results:**

Five overarching themes were identified: (1) public health infrastructure; (2) disease prioritisation and surveillance; (3) resource allocation and funding challenges; (4) collaboration and partnerships; and (5) direct impacts of COVID‑19 on NTD activities. Participants reported that COVID‑19 exposed and intensified pre‑existing weaknesses in health system capacity, routine NTD surveillance, and financing, contributing to delays and reduced coverage of mass drug administration (MDA) and other interventions. Donor funding reallocation and the withdrawal or scaling back of some international partners created further uncertainty for NTD programmes, although various national institutions and the World Health Organization (WHO) helped sustain essential activities. At the same time, the pandemic period coincided with the strengthening of the Zambia National Public Health Institute and the development of updated NTD and One Health strategic plans. Stakeholders viewed these institutional changes as opportunities to build a more resilient NTD response.

**Conclusions:**

COVID‑19 disrupted NTD programmes in Zambia primarily by amplifying long‑standing structural vulnerabilities in funding, surveillance, and service delivery, rather than through complete programme collapse. Strengthening routine NTD data systems, securing more predictable domestic financing, operationalising One Health and NTD master plans, and building more resilient, locally owned partnerships will be essential to recover lost ground and protect NTD control efforts from future shocks.

**Supplementary Information:**

The online version contains supplementary material available at 10.1186/s40249-026-01491-0.

## Background

Neglected tropical diseases (NTDs) are diseases of poverty that disproportionately affect populations in low‑ and middle‑income countries and are a key focus of the World Health Organization’s (WHO) 2030 roadmap for control and elimination [1]. Zambia is a land‑locked lower‑middle‑income country in southern Africa where multiple NTDs, including schistosomiasis, lymphatic filariasis, soil‑transmitted helminths, trachoma and rabies, remain endemic and contribute substantially to morbidity among communities with high levels of poverty and limited access to safe water and sanitation [2, 3].

Before the COVID‑19 pandemic, Zambia had already initiated mass drug administration (MDA) programmes and other control activities for several NTDs and had begun to consolidate national efforts through an NTD Master Plan and wider health‑sector reforms. However, as in many low‑ and middle‑income settings, these programmes operated within a context of chronic health‑system constraints, including workforce shortages, weak surveillance systems, and dependence on external partners for funding and implementation. Global evidence now shows that COVID‑19 has disrupted NTD programmes through interruptions to MDA, reductions or reallocation of financial resources, and delays in research and service delivery, with potential implications for progress towards 2030 targets [4–7].

Zambia experienced multiple waves of COVID‑19 and associated public‑health measures, alongside the development of new strategic frameworks, including the 2022–2026 NTD Master Plan and the National One Health Strategic Plan [8]. This combination of pandemic shock and policy renewal makes Zambia an important case for understanding how national NTD programmes were affected, and how they might be strengthened in the post‑pandemic period. This qualitative study therefore aimed to explore how national‑level stakeholders perceive the impact of COVID‑19 on NTD programmes, funding, surveillance, and partnerships in Zambia, and to identify opportunities to enhance the resilience of NTD control efforts in the years ahead.

## Methods

### Study design and setting

We conducted a qualitative case study to explore how the COVID‑19 pandemic affected NTD programmes in Zambia, with a focus on policy and programme decision‑makers. Zambia is a lower‑middle‑income, NTD‑endemic country that experienced multiple waves of COVID‑19 alongside long‑standing health system constraints.

### Sampling and recruitment

We used purposive sampling to identify key informants with direct experience in planning, financing, or implementing NTD‑related activities in Zambia during the COVID‑19 pandemic. Recruitment of participants took place between 01 March 2023 and 01 August 2023. Potential participants were identified through existing professional networks and recommendations from national experts working in NTDs and other related public health programmes. Individuals were eligible if they held a senior or technical role in government, multilateral agencies, non‑governmental organisations(NGO), or academia and were involved in NTD policy, programming, or surveillance between 2015 to 2023; frontline health workers without strategic responsibility were not targeted for this study.

A total of 18 key informants were approached, of whom 14 agreed to participate and completed an interview. Participants included national‑level Ministry of Health officials, staff from the Zambia National Public Health Institute, representatives of international organisations supporting NTD activities, and academic researchers with NTD expertise. One interview was conducted in a small group format with seven participants from the veterinary sector; because they spoke collectively about shared departmental experience, their contributions were analysed and reported as a single key informant to protect anonymity. This arrangement reflected a departmental decision and time constraints on the part of the subjects, and because participants spoke collectively about shared departmental experience (Table 1).  During analysis the transcript from this discussion was first coded at the level of individual turns of talk where distinct perspectives could be identified. For reporting, however, the material was treated as a single group case. This approach allowed potentially divergent views within the discussion to inform coding while recognising that reporting the interview as a single informant may have obscured some individual differences.

**Table 1:** Participants' affiliation

Offices involved in interviews	Number of people interviewed
Zambia National Public Health Institute	1
Ministry of Health	1
Lusaka Civil Council	1
Food and Agriculture Organization	1
WHO	1
Tropical Disease Research Centre	1
University of Zambia	1
Department of Veterinary Services	7

### Data collection

Data were collected using semi‑structured interviews guided by an interview schedule developed by the research team based on existing literature and prior work on COVID‑19 and NTDs. The guide covered participants’ roles, perceived impacts of COVID‑19 on NTD service delivery and MDA, funding and partnerships, surveillance, and broader health system functioning. The semi-structured interview guide was developed de novo by the lead author based on the study objectives and a review of the NTD and COVID-19 literature. Before use, the guide was reviewed within the research team and discussed with Zambian collaborators familiar with the policy and programme context to assess clarity, relevance, and coverage of key domains. Its content was subsequently compared with existing tools; the guide was not formally piloted prior to data collection. The interview guide will be provided as Additional File 1 to enable readers to assess the breadth and focus of each question.

Interviews were conducted in person by the lead author, a researcher with training and experience in qualitative methods and NTD research, at the participants’ workplaces or other private locations. Interviews were conducted in English, lasted approximately 30–60 min, and were audio‑recorded with participants’ consent. Verbal and written informed consent were obtained from all participants prior to the interview.

### Transcription and data management

Audio recordings were assigned unique study identifiers, stored on a secure, password‑protected server at the University of Edinburgh, and accessible only to the research team. The recordings were manually transcribed verbatim by the authors and cross‑checked against the original audio to ensure accuracy and fidelity to participants’ accounts. During transcription, all direct identifiers (such as the individuals’ names and specific institutions) were removed or replaced with generic descriptors, and each participant was assigned a pseudonym (Key Informant 1, Key Informant 2, etc.).

### Reflexivity and positionality

The lead author is a UK-based researcher with prior experience in NTD research and qualitative methods, while the wider team included collaborators with contextual knowledge of Zambia's health and veterinary sectors. This combination of external and locally informed perspectives shaped both access and interpretation: the lead author's position may have encouraged participants to explain institutional processes in detail, but it may also have influenced how senior officials framed programme successes, constraints, and relationships with international partners. To reduce the influence of any single perspective, interpretations were discussed within the multidisciplinary author team, coding decisions were reviewed collaboratively, and analytic memos were used to document assumptions and emerging interpretations throughout the analysis.

### Data analysis

We analysed the data using an inductive thematic analysis approach. First, the lead author read all transcripts several times to achieve familiarisation and made initial analytic notes on emerging ideas. Transcripts were then coded manually, line‑by‑line, to identify segments of text related to the research aim, allowing codes to be generated directly from participants’ language rather than from a pre‑existing framework.

An initial coding framework was developed from a subset of transcripts and iteratively refined as new data were coded. A second researcher independently reviewed the coding and discussed areas of disagreement with the lead author; differences were resolved through discussion and, where necessary, by revisiting the data to reach a consensus on the code definitions and boundaries. Related codes were then grouped into higher‑order categories, and candidate themes were developed through constant comparison across transcripts. The final thematic structure comprised five overarching themes: public health infrastructure; disease prioritisation and, surveillance; resource allocation and funding challenges; collaboration and partnerships; and direct impacts of COVID‑19 on NTD activities. No qualitative software was used; instead, we maintained an audit trail of coding decisions and theme development in analytic memos to enhance transparency and trustworthiness.

During coding, we monitored whether new concepts were still emerging and observed that after approximately ten interviews, no substantively new codes were identified, suggesting that we were approaching thematic saturation for the national‑level stakeholder perspectives included in this study while recognising that saturation cannot be assumed for all stakeholder groups in Zambia (Table 2).

**Table 2:** Themes

Theme	Key focus
Public health infrastructure	Health system capacity for neglected tropical disease (NTD) delivery
Disease prioritisation and surveillance	Relative attention given to NTDs in policy and planning, Detection, reporting and monitoring of NTDs
Resource allocation and funding	Availability and distribution of financial resources
Collaboration and partnerships	Coordination among key stakeholders
COVID-19 impacts on NTDs	Disruption to NTD activities and services

### Ethical considerations

Ethical approval was obtained from the University of Edinburgh Medical School Research Ethics Committee (reference 23‑EMREC‑011; 11 July 2023) and the University of Zambia Biomedical Research Ethics Committee (reference 4747‑2023; 4 January 2024). Data collection took place in August 2023 after approval from the University of Edinburgh committee and under institutional permissions from the University of Zambia and Ministry of Health for the collaborative research programme; the University of Zambia Biomedical Research Ethics Committee subsequently provided retrospective approval for the protocol already implemented. All participants received written information about the study, provided written and verbal informed consent, and were reminded that participation was voluntary and that they could withdraw at any time prior to anonymisation of the data. Audio files and transcripts were stored on secure, access‑restricted institutional servers, and only de‑identified data were used in analysis and reporting. The study was conducted in accordance with relevant ethical guidance for research involving human participants, including the principles of the Declaration of Helsinki.

## Results

### Public health infrastructure

Key informants described an evolving health system within Zambia that has been gradually expanding access to care over the past two decades, with COVID‑19 highlighting the importance of further investment in workforce, infrastructure and preparedness. The Zambia National Public Health Institute, supported by the Ministry of Health and international partners, was viewed as a key development, signalling government commitment to stronger surveillance and pandemic readiness by providing a national focal point for One Health approaches (Table 2).

Key informants from the veterinary sector emphasised that animal inspections for human consumption safety are conducted daily by registered veterinarians employed by the city of Lusaka. These inspections are considered essential for preventing conditions such as taeniasis and cysticercosis. Post-mortem inspections in municipal abattoirs fall under the jurisdiction of the Civic Council and continued throughout the pandemic, including during lockdown, although information on animal health is reported upwards to regional offices rather than systematically shared between districts. When sick animals are detected, the Civic Council records the source farm and farm conditions, while also conducting follow-up visits from officers and veterinarians to assess whether neighbouring animals are affected. However, informants noted that COVID-19 indirectly disrupted this system because some farmers were reluctant to bring animals into Lusaka, opting instead to slaughter them in less regulated peri-urban settings, therefore impacting follow-up at the farm level and preserving other infected animals. As one key informant explained, “*our abattoirs are not performing well within the city, so what most of these businesses have done is they’ve gone into the outskirts”* (KI3).

Participants noted that integrated, One Health‑oriented activities can increase efficiency but are also resource‑intensive. As one informant explained, “*we have been trying to integrate, but it is also quite expensive, complicated because there are some costs that come with integration, drugs, other human resources, and the training of the health makers to help with the programme*” (KI2). KI3 also described how essential services such as veterinary inspection of meat continued during the pandemic, but some slaughter practices shifted to less regulated settings on the outskirts of the city, raising concerns that zoonotic infections could be missed while some farms also failed to receive follow-up. Overall, respondents felt that creating more unified public health infrastructure and coordination across sectors would strengthen Zambia’s capacity to deliver and sustain NTD interventions.

### Disease prioritization and surveillance

Key informants reported that NTDs have historically received limited priority in Zambia, reflecting constraints in funding, human resources and surveillance capacity. Participants emphasised that it is difficult to design effective control programmes if there is not sufficient evidence to show where the diseases are prevalent and how many people are impacted. As noted by several informants, the choice about which disease to address first was often driven by available funding, rather than potential population benefit. Several informants stressed the need for more proactive, nationwide surveillance of NTDs, a goal that is reflected in the 2022–2026 NTD Master Plan.

Stakeholders described recent efforts to elevate NTDs and zoonotic diseases within a broader One Health agenda, including a national zoonotic disease prioritisation exercise and the development of a One Health Strategic Plan. These initiatives aim to bring together human, animal, and environmental health sectors and to provide a clearer framework for which conditions should receive attention and resources. As one participant explained, “*when you have a prioritized list, it opens up a lot of doors… the minute we prioritize it, it means now we can pull resources, and find it, even from central government*” (KI1). Participants also linked prioritisation to climate‑related risks, noting that recurrent droughts and floods can influence vector populations and disease transmission, reinforcing the importance of integrated, forward‑looking surveillance and planning for NTDs. Partipicants suggested that stronger surveillance is important not only for documenting burden but also for shaping programme eligibility, resource mobilsation, and continuity of control activities during periods of system shock. 

### Resource allocation/funding challenges

Key informants described chronic instability and insufficiency of funding for NTD programmes, which the COVID‑19 pandemic further exacerbated. Sustainable domestic financing and protected NTD budget lines were seen as essential, but largely lacking, with many activities reliant on a patchwork of government resources, project grants and external partners. One participant noted that even when national plans called for country‑wide MDA, “*we were supposed to do the mass drug administration countrywide, but we have just been funded to do almost half of the country*,” highlighting how funding gaps translate directly into reduced coverage (KI2).

Although medicines for several NTDs are donated through international partnerships, informants stressed that unfunded costs for transport, logistics, and community health worker support remain substantial. As one key informant explained, the government mainly contributes vehicles (for example, cars and trucks for transporting drugs to communities) and limited operational funds, meaning that “*funding is just a very minimal budget for the NTDs*” and the true cost of getting drugs from central stores to communities is often underestimated (KI2). Participants linked these constraints to the broader economic conditions in Zambia and donor countries, with one noting that declines in NTD funding began before the COVID‑19 pandemic and were “*a general reduction of funding… started back in 2014*,” (KI5). Potentially, further exacerbated by pandemic‑related economic downturn rather than explicit diversion of NTD budgets.

Several informants also noted that when major global initiatives cover specific programme costs, this can shrink the fund allocation available from agencies such as WHO; as KI6 explained, “*when I started many years ago, I used to have big budgets…the more funding we have like the END Fund and Global Fund, the smaller your budget gets because we don’t need as much WHO funding*” (KI6). At the same time, KI5 suggested that, as overall resources grow, “*issues of neglected diseases probably will start now, because when funding is too low, the prioritization of diseases also comes into play, if you have excess money, or you have enough money at least some other you start to recruit other neglected questions*” (KI5). K15 highlights the importance of cautious optimism in light of recent prioritisation of zoonotic diseases and NTDs within national strategies, which could help unlock additional funding for NTDs amidst other priorities, such as current cholera outbreaks.

### Collaboration and partnerships

Key informants described a dense network of collaborations between national institutions and international partners in planning and delivering NTD interventions in Zambia. These partnerships included long‑standing support from international non-governmental organizations (NGO) and academic institutions, which have historically played a central role in MDA and technical assistance. Participants noted that during COVID‑19, several NGOs reduced or paused their in‑country activities, leaving gaps in ongoing control programmes and contributing to delays in planned interventions.

Government agencies and the Zambia National Public Health Institute were reported to have taken on greater responsibility, with support from the WHO to sustain essential activities. Informants highlighted WHO’s role in providing technical support and helping to ensure that key MDAs, such as those for lymphatic filariasis and schistosomiasis, eventually proceeded despite earlier disruptions. At the same time, some partners withdrew or scaled back support: one respondent observed that after COVID‑19 began, “*we saw these partners [NGOs] pulling out… because they were complaining that their funding now has been channelled to support the COVID programme in their various countries*” (KI2). Although a few organisations, such as the International Trachoma Initiative, continued to operate with reduced funding, participants felt that the loss or reduction of external partners disrupted NTD elimination efforts, particularly those programmes that depended on several consecutive years of MDA.

### Impact of COVID-19

Key informants reported that the impact of COVID‑19 on NTD‑related work varied across sectors: some experienced little disruption, while others described major delays linked to travel restrictions, supply chain problems and shifting priorities. Overall, most participants felt that although programmes did not collapse, the delays and the alterations to the programmes remain noticeable in 2023.

Delays were common where activities depended on international staff or imported commodities. One informant explained that “*a lot of things were delayed because you couldn’t bring things into the country… flights were affected, personnel, people scaled down a lot of things*,” which slowed progress towards elimination (KI4). Rather than cancelling NTD activities, programmes were postponed or modified; for example, the delivery of malaria bed nets was adapted to comply with COVID‑19 precautions, but “*instead of doing what you would normally do, it might take twice as long to get to the same number of houses*” (KI6).

For NTDs requiring repeated MDA, participants highlighted that gaps in implementation risked undermining control efforts. One informant noted that schistosomiasis MDA experienced a 2‑year delay and that, more broadly, “*we are just doing one intervention… the children go back to the water, they get infections, they come back, we treat, and so forth*,” underscoring how interruptions and limited intervention breadth can perpetuate transmission (KI2). At the same time, some interviewees felt that COVID‑19 had relatively limited practical impacts on their own work, reflecting differences in roles and the feasibility of remote or office‑based activities.

## Discussion

This qualitative case study indicates that the COVID‑19 pandemic disrupted NTD programmes in Zambia mainly by amplifying long‑standing structural weaknesses in funding, surveillance and health‑system capacity, rather than through complete programme collapse. Participants described how delays to MDA and other interventions, partner withdrawals, and reallocation of resources slowed progress towards NTD targets and are still being felt several years after the initial waves. At the same time, they highlighted important investments in public health infrastructure and governance during this period, including the strengthening of the Zambia National Public Health Institute and the adoption of updated NTD and One Health strategic plans, which could support a more resilient NTD response in the future. These findings can be interpreted through a health system resilience lens. Using the WHO health system building blocks as a broad organising framework, the disruptions described by participants spanned service delivery, health information systems, workforce capacity, financing, and governance. The study also aligns with the resilience perspectives that emphasise not only the ability to absorb shocks, but also the capacity to adapt and reorganise in response to crisis. In this case, Zambia's NTD response did not remain unchange; instead it appeared to adapt unevenly with some functions delayed or narrowed while others were sustained through institutional improvisation, partner substitution, and emerging strategic planning. The findings on weak routine NTD surveillance and limited prioritisation are consistent with evidence from other low‑ and middle‑income countries, where fragmented data systems and constrained analytical capacity undermine timely, data‑driven decision‑making. In Zambia, interviewees reported that COVID‑19 further diverted attention and resources away from NTDs, delaying mapping and monitoring activities, while narrowing the focus to a small number of conditions with established programmes. The recent NTD Master Plan and One Health Strategic Plan explicitly aim to integrate human, animal, and environmental surveillance and to ensure that NTDs and zoonoses receive greater policy attention and resource prioritisation, suggesting that the policy foundations for improvement now exist, even if implementation remains uneven. The funding and partnership findings also speak to the political economy of global health financing.  Reliance on external actors brought technical expertise, commodities, and delivery support, but also created exposure to funding priorities set elsewhere.  These findings underscore that the withdrawal or downsizing of international NGOs during and after COVID-19 has important implications for the long-term sustainability of NTD programmes, particularly where external partners have historically supported MDA and logistics. At the same time, the coexistence of Zambia’s One Health Strategic Plan with persistent sectoral silos in practice highlights a tension between integrative policy aspirations and the fragmented institutional and financing arrangements that continue to shape NTD control efforts. Accounts of municipal abattoir inspections illustrate how certain veterinary public health services were formally maintained as “essential” throughout the pandemic, yet still experienced indirect disruption through changes in farmer behaviour. Reduced use of official abattoirs and a shift towards slaughter practices in less regulated peri-urban settings potentially undermine the surveillance for zoonotic infections of cysticercosis and weaken the links between detected animal disease, farm-level follow-up, and broader One Health objectives.

Resource allocation and funding challenges emerged as a central theme. Informants described chronic underfunding, heavy reliance on external partners, and the vulnerability of NTD budgets to wider economic shocks. The pandemic period coincided with both domestic fiscal pressures and shifts in international aid, which together produced unstable and sometimes declining funding for NTDs. Participants emphasised that donated medicines alone are insufficient: the unfunded costs of transport, logistics, and community health worker support frequently limited the scale and continuity of interventions. These perspectives reinforce calls for more predictable domestic financing and protected NTD budget lines, alongside clearer planning for how external funds complement, rather than displace, national investment.

The study also highlights collaboration and partnerships as both a strength and a vulnerability within Zambia’s NTD landscape. Long‑standing relationships with NGOs, academic institutions, and multilateral agencies were seen as critical for achieving coverage and providing technical support. However, several partners reportedly reduced or paused activities during COVID‑19, leaving gaps that national institutions and the WHO had to fill. While this demonstrated adaptability and commitment from domestic actors and WHO, it also raised concerns about the long‑term sustainability of programmes that depend heavily on external funding and expertise. Strengthening national ownership, clarifying roles and expectations, and planning for continuity when partners withdraw will be important for future resilience.

Finally, participants described heterogeneous direct impacts of COVID‑19 on NTD‑related activities. Some sectors were able to adapt by modifying delivery strategies—for example, changing how bed nets were distributed—while others experienced substantial delays due to travel restrictions, staff redeployment and commodity shortages. For NTDs that require repeated rounds of MDA, informants were particularly concerned that interruptions and inconsistent coverage could undermine progress towards control and elimination. At the same time, a minority of interviewees felt the practical impact on their own work was limited, reflecting differences in role, sector and ability to work remotely. Together, these findings suggest that the pandemic acted as a stress test, revealing both vulnerabilities and areas of resilience within Zambia’s NTD response.

This study adds a country‑specific, stakeholder‑level perspective to the growing literature on COVID‑19 and NTDs. By foregrounding the experiences of decision‑makers and technical leads, it highlights how global shocks intersect with existing health‑system constraints, funding architectures, and partnership arrangements. The findings point to concrete opportunities for strengthening Zambia’s NTD programmes: investing in routine surveillance and data use, securing more reliable domestic financing, operationalising One Health and NTD strategic plans, and building partnerships that are robust to future crises.

### Limitations

Several limitations should be considered when interpreting these findings. First, interviews were conducted in 2023 about events that occurred primarily in 2020–2021, so participants’ accounts may be affected by recall bias and selective memory. Second, many informants held senior roles in government or partner institutions, which may have introduced social desirability bias, with respondents potentially reluctant to criticise aspects of the national COVID-19 or NTD response. We also acknowledge that conducting one interview in a small group format may have influenced what participants felt comfortable sharing, as group dynamics can shape who speaks and which perspectives are voiced. This is a single-country qualitative case study with a small, purposively selected sample of national-level stakeholders, which limits the transferability of findings to other settings and to frontline or community perspectives. Given the relatively small, purposive sample and the diversity of institutional roles, we cannot claim exhaustive saturation across all possible stakeholder perspectives in Zambia; instead, we aimed to capture major recurring themes among national‑level decision‑makers, which appeared to stabilise by the later interviews.

## Conclusions

This qualitative case study shows that the COVID-19 pandemic disrupted NTD programmes in Zambia primarily by amplifying pre-existing weaknesses in funding, surveillance, and health system capacity, rather than creating entirely new challenges. While MDA and other core activities were delayed and scaled back, national institutions and partners ultimately maintained essential services, and the period coincided with important investments in public health infrastructure, including the Zambia National Public Health Institute and updated NTD and One Health strategic plans [9].

Going forward, strengthening routine NTD surveillance, securing more predictable domestic financing, and translating One Health and NTD master plans into fully implemented, multi-sectoral programmes will be critical to recover lost ground and protect NTD control from future shocks. Ensuring that partnerships are resilient, locally owned, and less vulnerable to external funding volatility will be equally important if Zambia is to sustain progress towards its NTD elimination targets in the post-pandemic era.

## Supplementary Information

Additional file 1.

## Data Availability

The datasets generated and analysed during the current study are not publicly available due to the risk of deductive disclosure among a small group of national‑level stakeholders, but de‑identified excerpts of transcripts may be made available from the corresponding author on reasonable request for academic purposes, subject to institutional approvals and data‑sharing agreements.
