# Supplementary material for: Impact of COVID-19 on neglected tropical diseases in Zambia: a qualitative study of stakeholder perspectives
Source: Infect Dis Poverty. 2026 Aug 25;15:93. doi: 10.1186/s40249-026-01491-0 (PMC13505077; doi:10.1186/s40249-026-01491-0)
Supplement: Supplementary file 1 [file 40249_2026_1491_MOESM1_ESM.docx]

Zambia questions for Semi Structured Interviews

Proposal for Zambia work

My project involves semi-structured interviews with key members of the Ministry of Health, WHO representatives of Zambia, FAO representatives in Zambia as well as any NGO organizations willing to talk with me. The goal of these interviews is to ascertain the impact of Covid on NTDS in Zambia, as well as the overall impact of NTD intervention programs running in Zambia pre and post covid. This will be used as a case study for my doctoral thesis as well as the basis for future work by comparing the impacts of Zambia against the impacts in other countries.

Confirm consent to participate and to be audio recorded.

1) Pre covid NTD Interventions:
   a. How did you think things were going in Zambia in relation to the SDG and NTDS prior to Covid?
      i. Overall, do you think NTDS in Zambia are in a better position than they were before the Millennium development goals and the beginning of the WHO list of NTDs?
   b. Do you think you were on track to meet the 2030 roadmap goals for NTDs?
   c. What interventions do you think were the most successful and why?
   d. What interventions were less successful and why?
   e. Before Covid- how would you rate the robustness of the healthcare system in Zambia?
      i. Were there doctor and nurse shortages before covid?
      ii. Were there medical supply shortages before covid?
2) Covid:
   a. What were Covid lockdown conditions like in Zambia?
      i. For how long? What public spaces/ essential services were opened, and which were closed?
   b. What impact do you feel those lockdown/restrictions had on the NTD interventions running in country?
   c. What (if any) was the impact on the economy of Zambia?
      i. Was a significant portion of the population unable to work due to restrictions or was there a large increase in people needing assistance? (Rewrite this, look and see if you can get any numbers on poverty in Zambia to rework this question)
   d. Were interventions still operating during the Covid pandemic? What delays did interventions face?
   e. How much of Zambia’s population is vaccinated against covid? (Current is 47% fully vaccinated as of dec 2022/ What is the popular opinion in Zambia of the covid vaccines?
   f. Do you think that the covid pandemic affected the Zambian population’s trust in medical officials or NGO workers?
3) Funding:
   a. What is the division between governmental funding for NTD interventions and donation/NGO funding for interventions in the country?
   b. Who decides in the country how the money that is donated is spent?
   c. How does governmental spending for NTDs get decided?
   d. Where do most donations of money and medicines in Zambia get used?
